# Addressing the challenges of regulatory systems strengthening in small states

**DOI:** 10.1136/bmjgh-2019-001912

**Published:** 2020-03-02

**Authors:** Charles Preston, Murilo Freitas Dias, José Peña, María Luz Pombo, Analía Porrás

**Affiliations:** Unit of Medicines and Health Technologies, Health Systems and Services, Pan American Health Organization/World Health Organization, Washington, District of Columbia, USA

**Keywords:** regulatory systems strengthening, reliance, regionalisation, medicines, small states

## Abstract

Countries should ensure equitable access to quality medicines. Regulatory systems for medicines and other health technologies are an essential part of well-functioning health systems and are a requisite for achieving Universal Health and the Sustainable Development Goals. The Pan American Health Organization, the World health Organization (WHO) regional office for the Americas, has assessed national regulatory capacities using a precursor of WHO Global Benchmarking Tool, and conducted an analysis of the data which suggests an association of regulatory capacity with population and the size of the economy. Regulatory capacity tends to decrease as population and gross domestic product decreases. This predominantly impacts the Caribbean sub-region in the Americas, which includes many states with small populations and economies. This paper will use the World Bank’s term ‘small states’ to refer to countries with 1.5 million people or less and other larger countries that face similar challenges. The regulatory challenges of small states include small markets and limited human and financial resources. However, small states can build regulatory systems with a narrower scope that are less resource intensive and still ensure appropriate regulation and oversight. The approach should be tailored to accomplish a subset of WHO recommended essential functions, including marketing authorisation, licensing of establishments and postmarket surveillance/pharmacovigilance, depending on the need to oversee local manufacturing, which requires a comprehensive system. The approach should also include adoption of efficiencies, such as regionalisation and reliance. This model is currently being put in practice in the small states of the Caribbean Community and Pacific Islands and can inform other small states around the world.

**Summary box**

- Regulatory systems are critical for ensuring safety, efficacy and quality of medicines and other health technologies.
- Yet data and analysis show that countries with small populations and gross domestic products face challenges in developing their systems that are unique.
- Pan American Health Organization proposes an approach to regulatory system strengthening in these small states that can help them accomplish the most important functions and, and do so more efficiently.
- The recommendations are applicable to small states around the world.

## Introduction

Countries should ensure equitable access to quality medicines. Regulatory systems for medicines and other health technologies are an essential part of well-functioning health systems and there is increasing focus on strengthening regulatory systems as a requisite for achieving Universal Health and the Sustainable Development Goals.^1 2^ A regulatory system is a government’s responsibility and should be an integral part of a national health system that operates within the context of defined pharmaceutical policy/legal frameworks. The Pan American Health Organization (PAHO), World Health Organization (WHO) regional office for the Americas, recommends that regulatory systems perform several essential functions (see section on recommended approaches for egulatory systems strengthening in small states). Systems unable to implement key functions are more vulnerable, and when they are weak or fail, they can result in injury or death of patients. However, health and regulatory systems making efforts to expand access to medicines that are safe, effective, quality-assured, and affordable face a convergence of challenges against a backdrop of limited resources: the globalisation of manufacturing and supply chains, varying quality of medicine sources, and the proliferation of new and complex products. Thus, regulatory authorities must do more with less, making it imperative to adopt efficiencies, leverage the work of others, and collaborate across regulatory authorities and institutions to achieve better regulation and oversight of national medicines markets.

## Regulatory systems capacities

Regulatory capacities vary greatly across different jurisdictions. WHO estimates that around 51% (99/194) of countries have some elements of a regulatory system but do not have a formal approach.^3^ Another 23% have systems that are evolving but that partially perform recommended functions in a reactive fashion. Approximately 26% have either stable, well-functioning and integrated systems, or systems operating at an advanced level of performance and continuous improvement (50/194). These categorisations are mostly based on the results of national regulatory assessment exercises conducted by WHO using the Global Benchmarking Tool (GBT), a comprehensive indicator-based tool that is used to assess regulatory capacities.^4^

The GBT assesses country capacities to perform the following functions: registration/marketing authorisation; licensing of establishments; market surveillance and control; vigilance; clinical trials oversight; regulatory inspections; laboratory testing and lot release of vaccines. For scoring, the GBT uses maturity levels, on a scale of 1–4, with 1 signifying the existence of some elements of a regulatory system, and 4 demonstrating an advanced level of performance and continuous improvement. Importantly, the assessment provides an objective input for the adoption of institutional development plans (IDP) to improve regulatory capacities and facilitates the monitoring of its implementation.

Since 2009, PAHO has been assessing national regulatory capacities using a precursor of the GBT^5^ that covers mostly the same functions and using a similar methodology. The tool consists of indicators deemed critical, necessary and informative. This voluntary process includes a PAHO-led peer-reviewed evaluation of the national regulatory system. While the most comprehensive methodology consists of an on-site evaluation, PAHO has also promoted abbreviated processes using a select set of indicators and/or assisted self-evaluations both as preliminary benchmarking and as IDP monitoring. To date, PAHO has conducted assessments of 27/35 (77%) member states.

**Figure 1:**
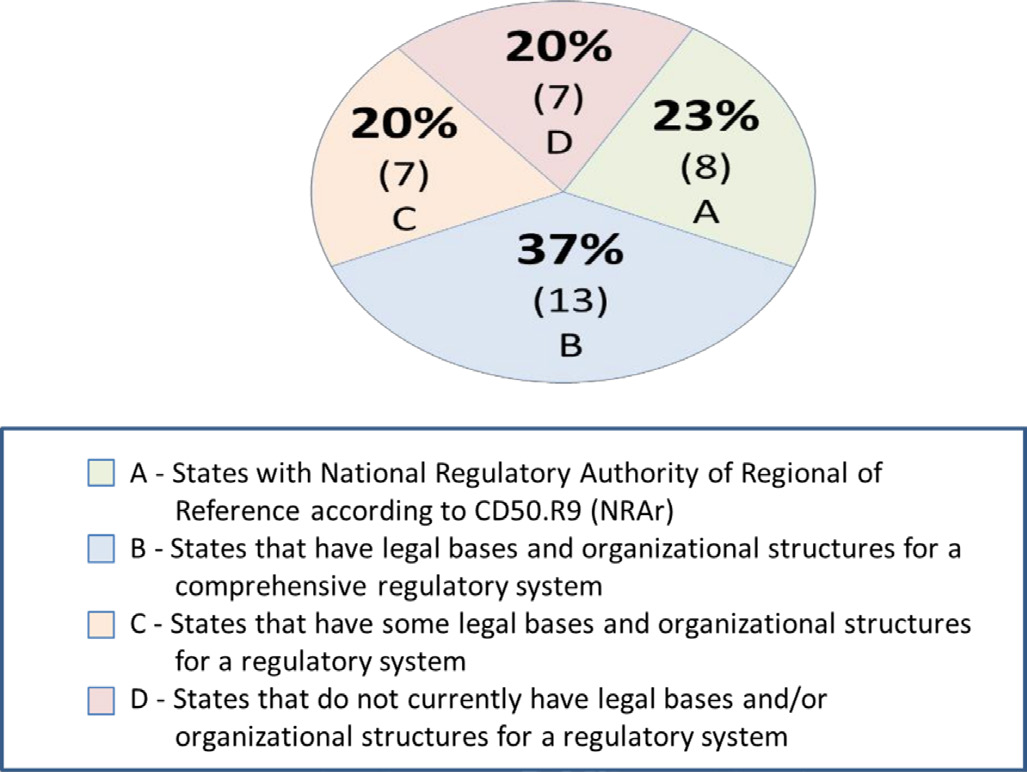
National regulatory capacity in the Americas (N=35).

Based on the assessment results conducted by PAHO, this paper groups countries into four categories according to the degree to which they demonstrated the legal foundations and organisational structures to operate a well-functioning system, similar to the WHO characterisation mentioned above. Where on-site comprehensive or abbreviated assessments were not possible, PAHO used official data such as pharmaceutical country profiles,^6^ combined with in-country knowledge to categorise the level of development of a national regulatory system. Figure 1 shows that 23% (8/35, group A) have achieved the highest level of development, which PAHO terms National Regulatory Authority of Regional Reference (NRAr).^7^ Another 37% (13/35, group B) have at least partially implemented^8^ all recommended functions towards a comprehensive system. An additional 20% (7/35, group C) of countries do not perform at least one of the recommended functions, and the remaining 20% (7/35, group D) fail to perform multiple functions, and are missing the requisite legal bases and/or organisational structures for regulatory systems.

**Figure 2:**
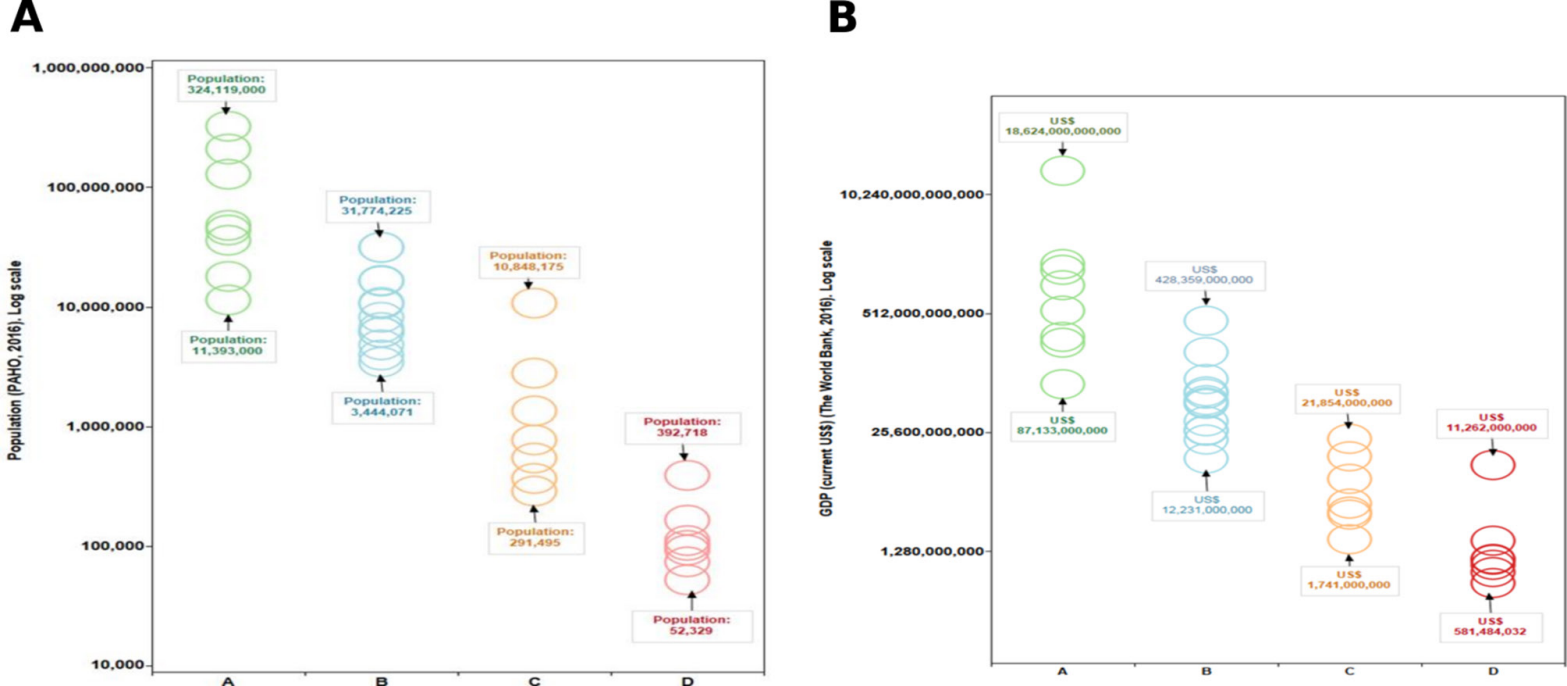
Regulatory system capacity based on population and GDP in the Americas (N=35). GDP, gross domestic product; PAHO, Pan American Health Organization.

Comparing the regulatory capacity data with population and economic information from the World Bank (WB)^9 10^ suggests an association of regulatory capacities with size of the population, and size of the economy, measured by gross domestic product (GDP). Figure 2A, B shows an association between decreasing regulatory capacity and smaller populations, as well as decreasing regulatory capacity and smaller GDPs. Regulatory capacity, however, does not seem to correlate with country income status (ie, GDP per capita) since many of the countries in groups C and D are considered either upper-middle or high-income by the WB. Absolute numbers of resources, both human and financial, are important for regulatory systems. This association suggests that a small population and small overall economy can negatively impact the human and financial resource requirements to operate systems effectively.

## Challenges of regulatory systems strengthening in small states

The data also highlight that in the Americas, 82% of the roughly 1 billion people live in countries with an NRAr. Another 16% live in countries that possess the legal and organisational elements for comprehensive systems, leaving 2% of the population with systems that have few or none of the legal and organisational foundations for regulatory systems. While this is a small percentage, it represents 18 million people, predominantly in the small countries of the Caribbean, living in health systems with rudimentary or no regulatory systems.

The Caribbean includes many states with small populations and economies, which for this paper will be referred to as ‘small states’. The term ‘small state’ references their affiliation with the WB’s ‘small state forum’ which gathers 50 countries that have challenges with economic development due to their size, including 12 Caribbean states.^11^ Of the 50, 42 meet the strict WB definition of population of 1.5 million or less, while the rest have larger populations but are part of the forum because they share similar challenges,^12^ related to economics, geography, remoteness and migration.

Small states’ regulatory challenges are in part influenced by the market dynamics in these countries. Small populations mean fewer consumers and smaller sales volumes. Some manufacturers, especially the largest ones, may choose not to operate in a market that is commercially unattractive. Geographical isolation and lack of proximity to larger markets may further decrease commercial incentives. Additionally, most of the commercial activity for medicines in small states is dominated by intermediaries, such as distributors and wholesalers, who may not be as prepared to face regulatory demands as large manufacturers. Hence, when small states move to increase regulatory standards, the intermediaries may be either unable or unwilling to comply, impacting the supply of medicines.

Small populations not only affect markets dynamics but may have an impact on the resources devoted to the regulatory system. In contrast to larger countries, the national health authority in small states tends to employ only a handful of people and have limited financial resources. Small populations can mean fewer people with competencies and experience to draw from. Human resources constraints can result in backlogs of products for marketing authorisation or there may be no designated official to receive and act on reports of adverse events and substandard/falsified medicines (SF). Some small states do not have any dedicated staff for regulating medicines and may only perform quality assurance activities linked to public procurement, with little or no oversight of the products sold in the private sector. In addition, due to a combination of low user fees and small public investment, regulatory systems in these countries tend to be severely underfunded and have difficulty securing the resources to accomplish tasks in an accountable manner.

## Recommended approaches for regulatory systems strengthening in small states

All states should assure the medicines that enter their health systems are safe, quality-assured and effective. Regulatory systems may regulate and oversee the products in their markets either directly or through collaborative efforts and other efficiency strategies. Any regulatory system, regardless of its size or context, should be guided by a series of basic principles, including independence, equity, transparency, ethical conduct, accountability and regulatory science. In addition, regulatory systems of any type or size should have a number of cross-cutting elements adapted to their specific context, such as financing, human resources and information systems.^13^ However, because regulatory systems can be resource intensive in human and financial terms, a major challenge is how to prioritise the recommended functions.^13^

In general, small states can build regulatory systems with a narrower scope, that are less resource intensive and still ensure appropriate regulation and oversight using a tailored approach to the needs of their health systems. The approach should focus on conducting a subset of WHO recommended functions and seek efficiencies to ensure they are performed adequately without compromising the basic principles and cross-cutting elements, or consuming too many resources whether financial or human. The subset of functions includes: (1) marketing authorisation, (2) market control/surveillance/vigilance and (3) licensing of establishments.

Since most if not all of the medicines entering small states are imported, small states should prioritise the control of importation and distribution practices, and thus, opt for conducting a streamlined marketing authorisation that aims to facilitate entry of quality products by relying on the oversight performed by regulatory authorities of trusted capacity. An abbreviated marketing authorisation should ensure safety, efficacy and quality by requesting key documents, such as the summary of product characteristics, electronic artworks (not physical samples), good manufacturing practice certificates and the certificate of pharmaceutical product (issued under the WHO scheme), to check that the product in question has marketing authorisation and is commercialised in a country with a trusted authority. The process should be able to establish that the product is exactly the same product approved by the trusted authority or WHO prequalification^14^ (ie, it is manufactured with the same active pharmaceutical ingredient, same standards, at the same site and on the same production line inspected by the trusted authority). Marketing authorisation should also focus on traceability of the product along its life-cycle.

Another critical area is market control/surveillance/vigilance, where even the smallest states should have a mechanism for spontaneous reporting by stakeholders, and preferably one that combines adverse event and SF medicines into one reporting form for efficiency. Furthermore, there should be human resources assigned to receiving these reports, to guarantee that the country readily detects and responds to problematic products on their markets. In countries that rely solely on imported products, licensing of importers and distributors and the warehouses from which they operate is of importance. Having local manufacturers introduces a high level of complexity to a small state’s regulatory strategy, however. because licensing and inspection of manufacturing facilities requires the implementation of additional and more complex regulatory functions. As a result, small states should decide if local manufacturing provides enough value to the health system to merit the establishment of a comprehensive regulatory system.

Other functions may not be critical. For example, there may not be a need to have clinical trials authorisation and oversight capacity if these do not occur in the country. Furthermore, although many states may think a drug testing laboratory is necessary, a national laboratory is not indispensable. Laboratories are very costly to operate, and require skilled human resources and infrastructure investments. Preregistration testing is a requirement in many countries but it is not considered a good practice since the laboratory test of an isolated sample is not informative of the overall quality of the product. Similarly, while laboratory testing is integral to postmarket surveillance programmes, a testing strategy should be risk-based and can be outsourced or shared with other trusted jurisdictions. Small states should have a well-established process to access laboratory facilities in suspected cases of SF medicines, and/or in the gathering of evidence for taking enforcement action.

As mentioned above, small states should not only focus on performing fewer and more streamlined functions but also adopt practices that can enhance efficiencies and scope in performing the recommended functions. Regionalisation;^15^ reliance/recognition^16 17^ and work sharing; fast-track/accelerated pathways; information sharing and the digitisation of systems can introduce efficiencies to the work of any regulatory system, but particularly benefit those with fewer resources at their disposal (table 1). Countries in the Caribbean Community (CARICOM) are implementing regionalisation through the Caribbean Regulatory System (CRS).^16^ Managed by the Caribbean Public Health Agency, it offers a single-entry portal for market authorisation to the 17 million people of CARICOM. Regionalisation increases market size, reduces fragmentation of standards and enables countries to conduct regulatory functions collectively or through individual members performing functions on behalf of others. It offers a chance for small states working together to achieve appropriate oversight of products circulating within CARICOM, where individually it would not have been possible.

**Table 1:** Efficiencies for regulatory system strengthening in small states

Efficiency	Description and benefits
Regionalisation	Occurs when countries or organisations with similar characteristics (eg, histories, cultural values, languages, etc) form collaborations to accomplish regulatory functions, usually through economic integration mechanisms. In regulation of medicines, this can result in a single portal of entry that increases market size, reduces fragmentation of standards and enables countries to conduct regulatory functions collectively or through individual members performing functions on behalf of others.
Reliance (including recognition)	The act whereby the NRA in one jurisdiction may consider andgive significant weight to—i.e. totally or partially rely upon—evaluationsperformed by another NRA or trusted institution in reaching its own decision.^17^ Recognition, a form of reliance, occurs when a regulatory system adopts the decision of another trusted entity. Relying on the work of other trusted regulators for regulatory functions (eg, marketing authorisation, inspections) may reduce staff workload and/or allow focus on other priority areas. Requires that the product under review has to be exactly the same as the one authorised and oveseen by teh trusted authority, including place(s) of manufacture.
Fast-track/ accelerated reviews	Many regulatory authorities employ mechanisms to speed the processing of products deemed of high public health value. These can occur with a reprioritisation of resources and focus or can be the result of shortened reliance-based processes Should not compromise product safety, efficacy or manufacturing quality standards.
Work sharing	A process, usually within the context of reliance, by which regulatory systems collaborate on regulatory activities such as assessing applications for marketing authorisation, joint work in postmarketing surveillance of therapeutic product safety, among others. Benefits include conserving staff and time resources, pooling scientific acumen and regulatory expertise, and so on.
Information sharing	A process whereby information on a variety of regulatory activities is shared either publicly or confidentially. It can be particularly helpful to share postmarket surveillance information among countries that have common products in their markets.
Digitisation	Digitising systems using commonly available software can provide benefits ranging from easier searching and organisation of records to decreased need for physical storage space. Allows for digital submissions that can attract off-site and foreign manufacturers. Use of a public website for the regulatory authority on the Ministry of Health webpage can be an inexpensive way to dramatically improve transparency and accountability, such as through publishing lists of approved products, licensed importers and enforcement actions.

Reliance on the marketing authorisations of trusted authorities as well as WHO prequalification, and/or foreign inspectional findings,^18^ can conserve valuable staff and financial resources on regulatory functions that larger and more established agencies are better equipped to perform. This in turn helps to fast-track or accelerate assessment timelines to months rather than years, which is the norm in most Caribbean countries. By improving timelines, small states can speed access to priority medicines for patients and increase commercial incentives for industry to operate in these markets. Although postmarket surveillance is one of the functions that needs to be performed locally to ensure that states gather information on how patients are tolerating medicines in local markets, small states can leverage international resources for more efficient postmarketing surveillance such as other authorities’ product alerts, WHO global databases for pharmacovigilance^19^ and SF medicines,^20^ regional networks for sharing information on products,^21^ and reliance on regional^22^ or WHO prequalified laboratories for product testing.

Digitising most regulatory processes, including receipt and review of marketing authorisation applications using commonly available software, can streamline numerous areas, such as searching and organising records and eliminating the need for physical storage space. Electronic filing may encourage manufacturers to work remotely increasing incentives to operate in geographically challenging areas. Field experience has shown that all countries, even the smallest and poorest in CARICOM, have information technology systems and websites. Putting in place a regulatory system webpage can be done very simply and cheaply as an addition to the existing Ministry of Health webpage. A regulatory website can dramatically improve transparency and accountability, through publishing a list of approved products and licensed importers, as well as recent regulatory decisions/enforcement actions, among others.

**Figure 3:**
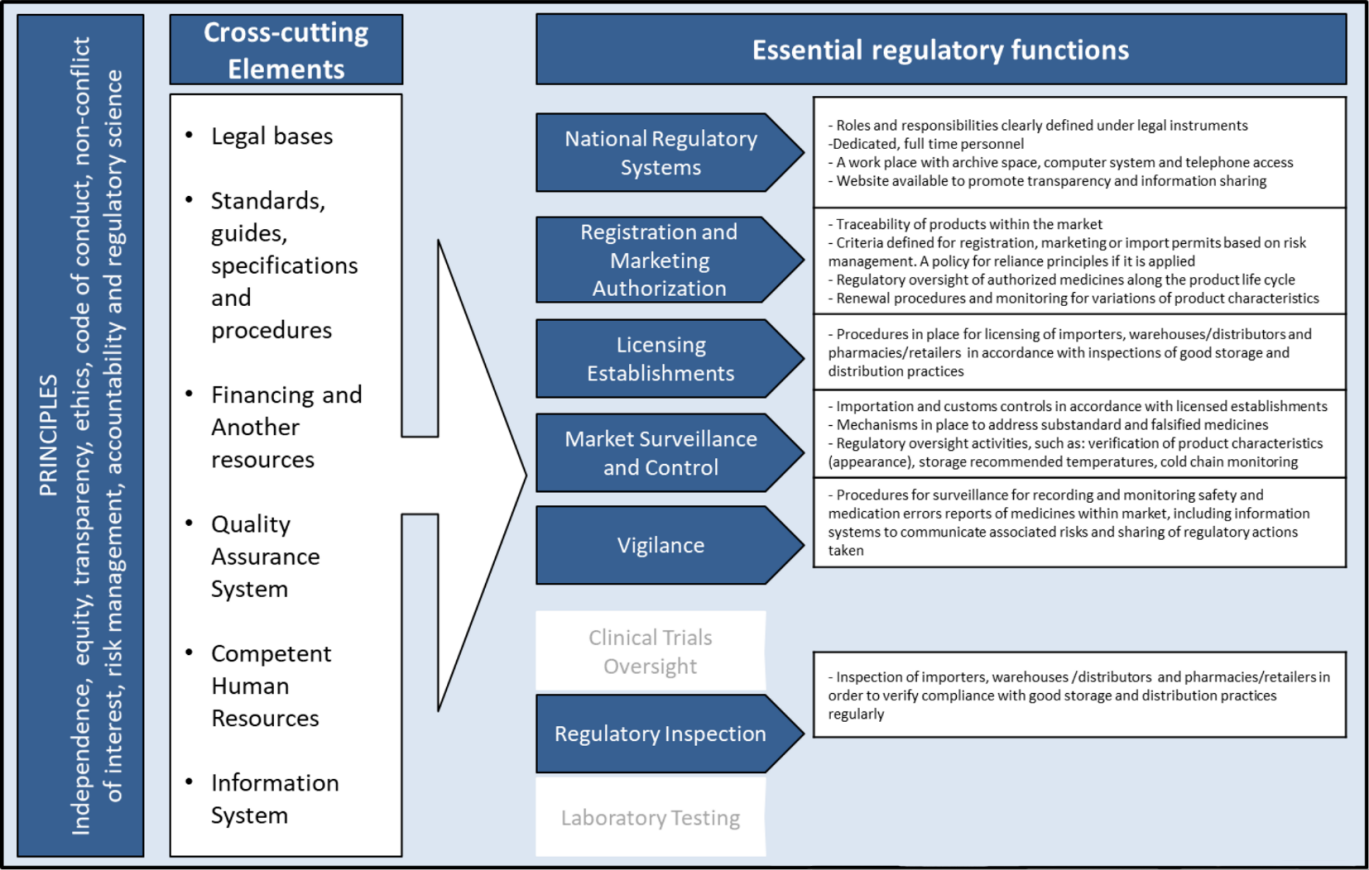
Essential functions recommended for small states. Source: Pan American Health Organization and Medicines and other Health Technology Unit/HSS.^13^

Figure 3 13 highlights in blue the recommended regulatory functions small states should prioritise. It is an adaptation of a regulatory framework developed and published by PAHO. The figure also details critical activities that small states should conduct within each recommended function.

## Conclusions

This article draws attention to the unique challenges faced by small states in the regulation of medicines and proposes a model to improve regulatory capacities and processes in these settings. It is informed by PAHO’s field experience in providing technical cooperation for strengthening regulatory capacities in the Americas, particularly, in the experience of supporting small countries and territories in CARICOM, and is based on the overall framework for regulatory system strengthening recommended by WHO. It is worth noting that most of the analytical work and establishment of the CRS precede the adoption of the GBT by the Americans. The GBT will further support the monitoring and evaluation of regulatory system strengthening strategies and help refine the recommendations for countries that aim to develop their regulatory capacities. Moreover, the GBT will eventually allow the assessment of integrated multi state systems such as the CRS. While small states may have a hard time achieving the maturity level 3 target individually, the CRS and its regionalised system may help boost their performance using this tool.

Regionalisation for small states is gaining traction on other continents. The small states of the Pacific Islands recently took a decision to implement regionalisation.^23^ Moreover, while the focus and recommendations of this paper are most relevant to small states, low-income countries that do not fit the small state definition but that struggle to devote sufficient resources to improve their regulatory capacities can also benefit from these recommendations. Similarly, these recommendations may apply to the regulation and oversight of other health technologies such as medical devices. A dual approach based on the implementation and strengthening of a subset of recommended regulatory functions and the adoption of efficiencies such as regionalisation; reliance/recognition and work sharing; fast-track/accelerated pathways; information sharing and digitisation can improve sovereign control of national medicines and other health technologies markets.
